# Vitamin Status and Diet in Elderly with Low and High Socioeconomic Status: The Lifelines-MINUTHE Study

**DOI:** 10.3390/nu12092659

**Published:** 2020-08-31

**Authors:** Yinjie Zhu, Isidor Minović, Louise H. Dekker, Manfred L. Eggersdorfer, Sander K.R. van Zon, Sijmen A. Reijneveld, Jenny E. Kootstra-Ros, Ido P. Kema, Stephan J.L. Bakker, Gerjan J. Navis, Ineke J. Riphagen

**Affiliations:** 1 Department of Internal Medicine, Division of Nephrology, University of Groningen, University Medical Center Groningen, 9713 GZ Groningen, The Netherlands; l.h.dekker@umcg.nl (L.H.D.); s.j.l.bakker@umcg.nl (S.J.L.B.); g.j.navis@umcg.nl (G.J.N.); 2 Department of Laboratory Medicine, University of Groningen, University Medical Center Groningen, 9713 GZ Groningen, The Netherlands; i.minovic@umcg.nl (I.M.); j.e.kootstra@umcg.nl (J.E.K.-R.); i.p.kema@umcg.nl (I.P.K.); i.j.riphagen@umcg.nl (I.J.R.); 3 DSM Nutritional Products, 4303 Kaiseraugst, Switzerland; m.l.eggersdorfer@umcg.nl; 4 Department of Health Sciences, University of Groningen, University Medical Center Groningen, 9713 GZ Groningen, The Netherlands; s.k.r.van.zon@umcg.nl (S.K.R.v.Z.); s.a.reijneveld@umcg.nl (S.A.R.)

**Keywords:** vitamins, socioeconomic status, elderly, nutritional status, diet quality

## Abstract

Socioeconomic health inequalities are an important global public health problem. However, it is not well known to what extent socioeconomic inequalities culminate in impaired vitamin status and whether this is mediated by diet. We, therefore, aimed to assess vitamin status in a population already at increased risk of micronutrient deficiency, i.e., elderly with high and low socioeconomic status (SES), and to investigate whether potential differences therein were mediated by diet quality. Vitamin status in 1605 individuals (60–75 years) from the Lifelines- Micronutrients and Health inequalities in Elderly (MINUTHE) Study was assessed by measuring folic acid and the vitamins B6, B12, D, A, E, and K. Multinomial logistic and linear regression analyses were applied to test the associations between SES and vitamin status. Mediation analysis was used to explore the interrelationship between SES, diet quality, and vitamin status. Low SES was associated with poorer status of vitamin B6, vitamin B12, and, notably, folic acid. Moreover, multivitamin deficiencies were more prevalent in the low SES group. Diet quality was found to mediate the associations of SES with folic acid (for 39.1%), vitamin B6 (for 37.1%), and vitamin B12 (for 37.2%). We conclude that low SES is a risk factor for a spectrum of vitamin deficiencies. Diet quality can partially explain the socioeconomic differences in vitamin status, suggesting that policymakers can mitigate socioeconomic inequality in nutritional status through improving diet quality.

## 1. Introduction

Socioeconomic status (SES) has been linked to health inequality worldwide, with low SES being one of the modifiable determinants of morbidity and premature mortality [1,2,3]. Meanwhile, international health strategies such as the Global Action Plan for the Prevention and Control of Non-communicable diseases (NCDs) failed to target socioeconomic inequality as a risk factor [4], and some public health interventions may even have further widened the health inequality gap [5]. 

Diet, as one of the most important health-related behaviors, has been well-documented to underly socioeconomic health inequality [6,7]. It is known that overall diet quality is worse in low SES groups because of less consumption of healthy foods (i.e., fruits and vegetables) and more consumption of unhealthy foods or macronutrients (i.e., sugar and processed meats) [6,8,9,10]. However, the effects of socioeconomically patterned differences on vitamin status have not been solidly characterized, since vitamin status has mainly been assessed by using food frequency questionnaire and food diaries, which are not suitable measures to assess vitamin status [11], or incompletely by measuring limited numbers of biomarkers of vitamin status [11,12,13,14]. 

Diet-induced differences in vitamin status may be especially relevant in the elderly population. The reason for this is that during aging, a decreased energy need results in lower consumption of foodstuffs, while most of the micronutrient requirements, including vitamin requirements, remain unchanged. Consequently, the elderly are prone to developing clinical and subclinical nutritional deficiencies [15,16]. Therefore, our primary objective was to comprehensively compare vitamin status between low and high SES in elderly, by measuring a spectrum of vitamin biomarkers, including folic acid, vitamin K, B12, B6, E, A, and D. Secondly, we aimed to investigate whether diet mediated the association between SES and vitamin status. 

## 2. Methods

### 2.1. Study Design and Population

The ongoing Lifelines Cohort Study was developed as an observational three-generation population-based follow-up study that aimed to reveal the relations and interactions between genomic and environmental determinants in the process of aging. A detailed description of the Lifelines Cohort study can be found elsewhere [17,18,19]. In brief, a total of 167,729 residents from Northern Netherlands were included to form a cohort that is representative of the Dutch general population [19]. People with severe psychiatric or physical illness, with limited life expectancy (<5 years), and those with insufficient knowledge of the Dutch language were not eligible and excluded from the study. Participants were physically examined and various bodily materials, including plasma, serum, and 24-hour urine, were collected and stored in the Lifelines Biobank. Furthermore, adult participants (≥18 years) were asked to complete several questionnaires regarding various aspects, such as demographics, the socioeconomic condition, and diet. All participants gave their informed consent for inclusion before they participated in the study. The study was conducted in accordance with the Declaration of Helsinki, and the protocol was approved by the Medical ethical committee of the University Medical Center Groningen Institutional Review Board, The Netherlands (2007/152). 

The MINUTHE (Micronutrients and Health inequalities in Elderly) study is a sub-cohort study from the Lifelines Biobank designed to investigate the interrelationship of SES, nutritional status, and health inequalities among elderly. For the MINUTHE study, 1605 participants aged between 60 and 75 years, who had available plasma, serum, and 24-hour urine samples, were selected from the Lifelines Biobank. The selection was performed at both extremes of the SES distribution while taking into account gender-balance, so to create four equally-sized groups: 400 men and 403 women with low SES, and 402 men and 400 women with high SES (Figure S1). Since education is more differentiating than income in the Dutch population [20], classification of SES was based on education attainment. Low SES was defined as at maximum primary school, or completed lower vocational or secondary schooling. High SES was defined as completed higher vocational schooling or university education [21]. 

### 2.2. Measurements

Anthropometric measurements and blood pressure were measured by well-trained staff. Anthropometric measurements were measured without shoes, and body weight was measured to 0.1 kg by the SECA 761 scale (Seca GmbH, Hamburg, Germany); height was measured to 0.5 cm using the Frankfort Plane position by the SECA 222 stadiometer (Seca GmbH, Hamburg, Germany); and the waist and hip circumferences were measured to 0.5 cm by the SECA 200 measuring tape (Seca GmbH, Hamburg, Germany). Systolic and diastolic blood pressures were measured 10 times within 10 min, and each of the average values of the last three readings was used as blood pressure parameters. Blood pressure was measured by Dynamap PRO 100V2 (GE Healthcare, Freiburg, Germany) [18]. Body mass index (BMI) was calculated as body weight (kg) divided by height squared (m^2^). 

The BMI was additionally categorized into normal (BMI < 25 kg/m^2^), overweight (25 ≤ BMI < 30 kg/m^2^), and obese (BMI ≥ 30 kg/m^2^) [22].

Between 2006 and 2013, fasting blood and 24 hours urine samples were collected, processed, and stored at −80 °C until analysis in 2016, indicating that the serum and plasma samples had been in storage for 3–10 years. Vitamins with reliable biomarkers available were selected in our study and previous studies showed that the selected vitamins in our study remained stable in plasma and serum samples after 13–15 years, so the storage time is unlikely to have impact on the parameters [23,24]. Glucose and homocysteine were measured using enzymatic assays (cobas c-module), and total vitamin B12 and folic acid were measured using electrochemiluminescence immunoassays (cobas e-module, Roche Diagnostics GmbH, Mannheim, Germany). Vitamin K status was assessed by measuring desphospho-uncarboxylated matrix Gla protein (dp-ucMGP) using a dual-antibody enzyme-linked immunoassay (InaKtif MGP (IDS-iSYS) assay). Vitamin B6 was measured using the high-performance liquid chromatography (HPLC) method, described in detail elsewhere [25] Vitamin E, vitamin A, and 25-hydroxyvitamin D were measured using validated in-house liquid chromatography-tandem mass spectrometry (LC-MS/MS) assays, which had coefficients of variations of <10.4%, <3.6%, and <14.1%, respectively. Hemoglobin A1c (HbA1c) was measured by means of a liquid chromatography assay (Sysmex etc.). All assays were validated before use.

### 2.3. Definition of Vitamin Status

Serum folic acid, vitamin K, B12, B6, E, A, and D status were measured in this study, and the cutoff values applied for each vitamin to define deficiencies, insufficiencies, and sufficiencies are shown in Table 1. The multivitamin status was identified and categorized into deficiency or insufficiency of a single vitamin, deficiencies or insufficiencies of two vitamins, and deficiencies or insufficiencies of more than two vitamins. 

### 2.4. Dietary Assessment

During the baseline visit, a semi-quantitative self-reported food frequency questionnaire (FFQ) was used to assess the intake of 110 food items over the last month; this FFQ was developed and validated by Wageningen University [35,36]. Frequency categories ranged from ‘not this month’ to ‘6–7 days per week’, with an indication of the portion size and household measurements units. Energy, macronutrients, and alcohol intake were estimated from the FFQ by using the 2011 Dutch food composition database (NEVO) [37]. The reliability of the FFQ data was assessed according to the Goldberg cutoff, which was based on the ratio between reported energy intake and basal metabolic rate (EI/BMR). The basal metabolic rate was calculated with the Schofield equation [38]. Based on the Goldberg cutoff method, the participants with a ratio below 0.87 or above 2.75 were excluded during the dietary assessment [39]. In total, 299 participants with missing (*n* = 238) or unreliable dietary data (*n* = 61) were excluded from the analyses regarding the dietary assessment in the current study. 

To rank the relative diet quality, the lifelines diet score (LLDS) was developed and calculated based on the 2015 Dutch dietary guidelines, which is an evidence-based summary of the literature on food and chronic diseases [40]. A detailed description of the LLDS could be found elsewhere [41]. In short, the LLDS identified and categorized nine positive food groups (vegetables, fruit, whole grain products, legumes and nuts, oils and soft margarines, unsweetened dairy, coffee, and tea) and three negative food groups (red and processed meat, butter and hard margarine, and sugar-sweetened beverages). Subsequently, the intake of the food groups was presented in grams per 1000 kilocalories (kcal) and scored from 0 to 4 in quintiles. The highest score was assigned to the highest quintile of consumption for positive food groups and the lowest quintile of consumption for negative food groups. The sum of scores of the food groups formed the LLDS with a range from 0 to 48 [41]. 

Drinking behavior was estimated based on the FFQ data. Heavy drinking was defined as eight or more alcoholic drinks per week for women, and 15 or more for men [42].

### 2.5. Statistical Analyses

Statistical analyses were performed using IBM SPSS 25 (SPSS, Chicago Illinois, USA) and R studio version 1.1.383 (RStudio, Redmond, WA, USA). Nominal variables are presented as frequencies (n, (%)). Normally distributed continuous data were shown as mean ± standard deviation (SD). Non-normally distributed continuous variables were described as median (interquartile range [IQR]). A two-sided P < 0.05 was considered indicating statistical significance.

Baseline characteristics are presented as mean ± standard deviation (SD) for parametric data or median [interquartile range] for non-parametric distributes of the data for the overall study population and for subjects with low and high SES. Differences between the two SES groups were tested using the Student’s T-test, Mann–Whitney U test, and the Chi-Squared test for parametric, non-parametric, and categorical variables, respectively.

Multinomial logistic regression analyses were applied to investigate the association between SES levels (i.e., low versus high SES) and categories of vitamin status (i.e., deficiency and insufficiency versus sufficiency). Additionally, linear regression was conducted as sensitivity analyses to test the association between SES levels (low and high) and continuous levels of serum (folic acid, vitamin B12, vitamin B6, vitamin D, vitamin E, vitamin A, and vitamin K) biomarkers. For both logistic and linear regressions, SES was first independently included in the crude mode (model1). Subsequently, the model was additionally adjusted for age and gender (model 2); then smoking behavior (current, former, and never), and BMI were added as covariates to form model 3. 

**Figure 1:**
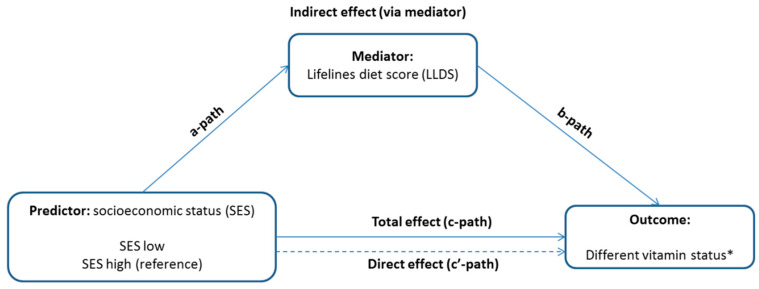
Conceptualized model of the Lifelines Diet Score as a mediator of the relationship between socioeconomic status (SES) and different vitamin status. * Vitamin status includes single- and multi-vitamin deficiency and insufficiency, and serum concentrations of the following vitamins: folic acid, vitamin B12, vitamin B6, vitamin D, vitamin E, vitamin K.

Next, as we wanted to explore the potential mediation effect of diet, the LLDS, daily vegetable intake, and fruit intake were firstly treated as covariates in model 4, model 5, and model 6, separately. Secondly, the LLDS was considered as a mediator between SES and vitamin status (Figure 1) and examined using the “Lavaan” package in R studio [43]. The mediation models consisted of SES (cause), LLDS (mediator), and categorical and continuous variables of biomarkers of vitamin status (outcome). The bootstrapping method was used to verify the mediation effects, the SE (standard error) for direct and indirect effects were shown from 5000 times bootstrapping, the statistical significance of the paths was evaluated using an alpha level of 0.05, two-tailed. 

## 3. Results

### 3.1. Participant Characteristics, Vitamin Status, and Diet

Participant characteristics are shown in Table 2. The median age of the study population was 65 years old (Interquartile range [IQR] 62–69 years old), and the mean BMI was 27.0 ± 4.1 kg/m^2^. The mean energy intake per day was 1909.0 ± 6.2 kcal, and the LLDS was 23.9 ± 6.2 among the whole population. The prevalence of single-vitamin deficiency was 28.7%, while 55.7% of the participants suffered from at least one vitamin insufficiency. 

We found no differences in total daily energy and protein intakes between low and high SES (*p* > 0.09 for both). However, the intake of carbohydrates and fats was higher in the low SES group, while alcohol consumption was higher among subjects with high SES (*p* < 0.03 for all) (Table 2). In addition, the LLDS was lower in the low SES group (i.e., 22.5 ± 6.2) compared to the high SES group (i.e., 25.1 ± 6.0; *p* < 0.001). The high SES group also had overall high intakes of foodstuffs that were generally perceived as healthy, such as vegetables, fruits, legumes and nuts, fish, unsweetened dairy products, and tea (*p* < 0.007 for all) (Table S1). 

**Figure 2:**
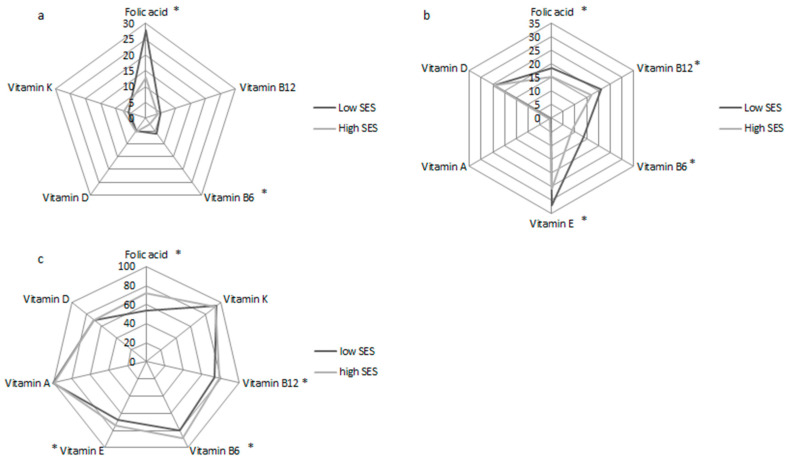
Prevalence (%) of vitamin deficiency (**a**), insufficiency (**b**), and adequate status (**c**) in low and high SES groups. * significant difference between low and high SES groups with *p* < 0.05. The cutoff values applied for folic acid status were serum folic acid < 10.2 nmol/L, 10.2–13.6 nmol/L, and > 13.6 nmol/L for deficiency, insufficiency, and sufficient status, respectively. Vitamin K insufficiency was defined as desphospho-uncarboxylated matrix Gla protein (dp-ucMGP) level higher than 500 pmol/L. The cutoff values employed for vitamin B12 status were serum vitamin B12 < 150 pmol/L, 150–220 pmol/L, and > 220 pmol/L for deficiency, insufficiency, and sufficient status, respectively. The cutoff values used for serum vitamin B6 status, were serum vitamin B6 < 20 nmol/L, 20–30 nmol/L, and > 30 nmol/L for deficiency, insufficiency, and sufficient status, respectively. Serum alpha-tocopherol levels below 30 μmol/L were considered indicating vitamin E insufficiency. Vitamin A insufficiency was defined as serum retinol concentration below 0.7 μmol/L. The cutoff values applied for vitamin D status, were serum 25-hydroxyvitamin D (25(OH)D) < 30 nmol/L, 30–50 nmol/L, and >50 nmol/L for deficiency, insufficiency, and sufficient status, respectively.

Moreover, we found significant differences in vitamin status between subjects with low and high SES (Figure 2). The high SES group showed higher B-vitamins (i.e., folic acid, vitamin B12, and vitamin B6) levels in their serum (*p* < 0.001 for all), while no difference was found among fat-soluble vitamins between low and high SES groups (*p* > 0.05 for all). Prevalence of vitamin E, vitamin B6, and folic acid deficiencies or insufficiencies were higher in low SES elderly (*p* < 0.01 for all). Low SES participants also had a higher prevalence of single and multivitamin deficiencies (*p* < 0.001) and insufficiencies (*p* = 0.006) compared with the high SES participants.

### 3.2. Associations between SES and Vitamin Status

The associations between SES and vitamin status are presented in Table 3 and Table S2. We found that elderly subjects with low SES had a higher risk for folic acid deficiency and insufficiency (*p* < 0.001 and *p* = 0.002, respectively), vitamin B6 deficiency (*p* = 0.001), and vitamin E insufficiency (*p* = 0.03) compared to those with high SES after adjustment for age, gender, BMI, and smoking behavior. Additionally, the low SES participants had a higher risk for single and multivitamin deficiencies compared with the high SES participants (*p* < 0.001 and *p* = 0.02, respectively). We found that low SES was associated with lower serum concentrations of folic acid, vitamin B12 and vitamin B6 after adjustment for age, gender, BM, and smoking behavior (*p* ≤ 0.007 for all) (Table S2).

### 3.3. The Mediating role of the LLDS in the Associations between SES and Vitamin Status

The mediation models of the LLDS in the associations between SES and vitamin status and serum biomarkers are presented in Table 4 and Table S3, respectively. The high SES group had higher LLDS than the low SES group (*p* <0.001); the increased LLDS was associated with higher serum folic acid (*p* < 0.001) and vitamin B6 (*p* = 0.02) levels, and lower risks of folic acid deficiency (*p* = 0.002), folic acid insufficiency (*p* = 0.01), vitamin B12 insufficiency (*p* =0.02), and vitamin B6 insufficiency (*p* = 0.006). The LLDS was found to partially mediate the associations between SES and folic acid deficiency (10.1%, *p* = 0.004), folic acid insufficiency (39.1%, *p* =0.03), vitamin B12 insufficiency (37.2%, *p* = 0.03), and vitamin B6 insufficiency (37.1%, *p* =0.01). When performing mediation analyses using serum vitamin biomarker concentrations, the LLDS was found to mediate serum folic acid (14.3%, *p* = 0.001), vitamin B6 (17.2%, *p* = 0.03), and vitamin A (27.7%, *p* = 0.04) concentrations.

## 4. Discussion

To the best of our knowledge, this is the first study to comprehensively describe vitamin status through measurements of a panel of vitamins (i.e., folic acid, vitamin B12, B6, A, D, E, and K) in elderly with low and high SES, and to comprehensively investigate the interrelationship between SES, diet quality, and vitamin status. We found that elderly with low SES had a significantly poorer vitamin status and that this difference could be explained, at least in part, by diet because the diet was a significant mediator of the status of folic acid, vitamin B12, vitamin B6, and vitamin A.

This study revealed that in home-dwelling elderly participants in the Northern Netherlands the prevalence of vitamin deficiencies and insufficiencies is relatively low over the whole range, with the exception of folic acid. Nevertheless, the overall prevalence of any vitamin deficiency is relatively high, and the prevalence of any vitamin insufficiency is considerable. Vitamin B12, B6, and D levels found in our population, were in line with previous studies performed in healthy elderly [29,44,45,46,47]. However, we observed a higher prevalence of folic acid deficiency compared to the recently published Irish study of Laird et al. [48], who revealed that folic acid status in older adults was most strongly determined by folic acid supplement use. Interestingly, the use of vitamin supplements in our study was lower compared to that of Laird et al., which may explain the higher prevalence of folic acid deficiency in our study population. Compared with literature conducted among adolescents and younger adults, we observed higher deficiency prevalence of folic acid [45], vitamin B6 [49], vitamin B12 [45], and vitamin D [47,50], further supporting the idea that elderly are at increased risk of developing nutritional deficiencies, compared to the general adult population [16,51]. However, proper comparison of our data with other studies is hampered due to the heterogeneous definition of suboptimal vitamin status, inconsistent age definition of elderly, and the plethora of different vitamin biomarkers used [27,47,50].

The intake of several important vitamins, including folate, vitamin B6, vitamin B12, vitamin D, vitamin A, and vitamin E, has been studied among different SES groups [9,12,52]. In accordance with previous literature, we found that elderly with a low SES had a significantly worse status of several important vitamins, compared to those with a high SES, indicating a quantitative difference [9,11,53]. In addition to those vitamins, vitamin K was included in our assessment in the context of SES for the first time. More importantly, none of the studies had incorporated objective measurements of those vitamins among different SES groups; thus, our study objectively validated the evidence from vitamin intake studies. While the SES difference of most vitamins has been limited to dietary resources, environmental factors, such as sunlight, are likely to influence the vitamin D difference as well [13,54]. 

In the present study, the difference in vitamin status between the SES groups was partially mediated by diet quality. The mediation effect was most pronounced for folic acid status, which may be explained by several factors. First, the low SES group consumed significantly less folate-rich foodstuffs, including fruits and legumes [8,9,10]. Second, unlike vitamin B12, which takes two to five years before its body stores are depleted [55,56], folic acid body stores are relatively limited. As such, inadequate intake can impair folic acid status within several months [54]. However, since the difference in folic acid, vitamin B12, and vitamin B6 status between the SES groups was not fully explained by diet quality, it is conceivable that other non-dietary factors are also involved. For instance, it has been shown that methylmalonic acid is a better predictor of vitamin B12 deficiency [57]. In addition, it has been postulated that inflammation may affect vitamin B12 and folic acid status. However, evidence for this hypothesis is somewhat limited and conflicting and requires further attention [58,59]. Other vitamins were not, or to a lesser degree, related to diet quality. In future studies, it would be interesting to investigate other pathways that are involved in SES and nutritional status such as sun exposure [54], physical activity [13,60], and malabsorption [61]. 

Our findings have provided support for the conceptual premise that hidden hunger is more likely to occur in the elderly population, particularly among low SES individuals [15]. Hidden hunger is a lack of vitamins and minerals whilst the energy intake remains sufficient and the causes of hidden hunger are multifactorial and include sufficient daily energy intake, high BMI, and prevalent suboptimal nutritional status, which may culminate in hidden hunger [62]. Since the risk factors for hidden hunger were significantly more pronounced in the low SES group, it is conceivable that this population is at an increased risk of adverse health effects and premature mortality. Consequently, the adverse health effects might inflict a greater societal need for care and a higher financial burden. Future studies should address these matters.

This study has several strengths. First, the selection of individuals was made from a large biobank that is representative of the Dutch general population [19]. Second, the large sample size of the biobank enabled maximum differentiation in SES. Third, by including comprehensive measurements of vitamin biomarkers, this study provided a more reliable assessment of vitamin status than many previous studies that used dietary intake data. However, there are several limitations that also need to be addressed. First, the cross-sectional study design prohibited a causal inference between SES, diet quality, and vitamin status. Second, the LLDS was initially developed with evidence on diet-disease relations, so some vitamin-rich food groups were not included (e.g., white unprocessed meat and cheese), which might decrease the representativeness of LLDS as an indicator of diet quality. 

It has been suggested that nutrition-promoting interventions should be tailored according to SES to minimalize the disproportionate health effect [5]. Although the elderly population was often addressed to be the vulnerable group [61], our study suggested that it was particularly important to address the low SES individuals among elderly. To promote healthy aging and ameliorate the socioeconomic health inequalities under the demographic shift towards an increasing elderly population [63], it is necessary to allocate resources equitably to improve diet quality and raise awareness of nutritional status among the elder population. 

In summary, this study revealed that low SES was a risk factor for suboptimal vitamin status (i.e., folic acid, vitamin B12, and vitamin B6) among the elderly. Importantly, suboptimal vitamin status was partly mediated by diet quality, highlighting the potential importance of diet in socioeconomically patterned health inequalities, and indicating the need for SES-tailored public health strategies. Further studies should assess other determinants of SES-related nutritional differences and explore whether these differences are related to clinically adverse outcomes.

## Figures and Tables

**Table 1:** Vitamin cutoff values.

Vitamins *	Vitamin status		
	Deficiency	Insufficiency	Sufficiency
Folic acid, nmol/L [26]	<10.2	10.2–13.6	>13.6
Vitamin K, pmol/L [27,28]	-	>500	≤500
Vitamin B12, pmol/L [26,29]	<150	150–220	>200
Vitamin B6, nmol/L [30,31]	<20	20–30	>30
Vitamin E, μmol/L [32]	-	<30	≥30
Vitamin A, μmol/L [33]	-	<0.7	≥0.7
Vitamin D, nmol/L [34]	<30	30–50	>50

* Vitamin K, vitamin A, and vitamin E status was defined from serum dp-ucMGP, retinol, and alpha-tocopherol concentrations.

**Table 2:** Demographics, diet, and vitamin status of the study population.

Characteristics	Total	Low SES	High SES	*p*-Value
	*N* = 1605	*N* = 803	*N* = 802	
**Demographics**				
Age, years	65 (62–69)	66 (63–70)	64 (62–67)	<0.001
Male gender, *n* (%)	802 (50)	400 (49.8)	402 (50.1)	0.9
BMI, kg/m^2^	27.0 ± 4.1	28.2 ± 4.2	25.7 ± 3.6	<0.001
Normal, *n* (%)	531 (33.1)	161 (20.1)	370 (46.1)	<0.001
Overweight, *n* (%)	749 (46.7)	410 (51.2)	339 (42.3)	
Obese, *n* (%)	323 (20.1)	230 (28.7)	93 (11.6)	
Smoking				0.3
Never, *n* (%)	547 (34.1)	264 (33.1)	283 (35.7)	
Former smoker, *n* (%)	853 (53.1)	429 (53.8)	424 (53.5)	
Current smoker, *n* (%)	191 (11.9)	105 (13.2)	86 (10.8)	
Waist/Hip ratio	0.94 ± 0.10	0.96 ± 0.08	0.93 ± 0.11	<0.001
Systolic blood pressure, mmHg	134 ± 18	137 ± 18	131 ± 17	<0.001
Diastolic blood pressure, mmHg	75 ± 9	76 ± 9	75 ± 10	0.09
**Diet**				
Lifelines diet score	23.9 ± 6.2	22.5 ± 6.2	25.1 ± 6.0	<0.001
Total energy, Kcal/day	1909.0 ± 518.0	1936.5 ± 548.3	1888.0 ± 493.0	0.09
Total carbohydrate, g/day	208.4 ± 62.1	216.5 ± 64.5	202.2 ± 59.5	<0.001
Total fat, g/day	74.7 ± 26.3	76.4 ± 27.6	73.3 ± 25.1	0.03
Total protein, g/day	71.8 ± 9.7	72.1 ± 19.4	71.6 ± 17.5	0.7
Total animal protein, g/day	42.7 ± 13.4	42.4 ± 13.6	42.9 ± 13.2	0.6
Total plant protein, g/day	29.3 ± 9.7	29.7 ± 10.2	28.9 ± 9.2	0.1
Percentage energy from:				
Carbohydrates	43.8 ± 6.3	44.9 ± 5.9	42.9 ± 6.5	<0.001
Protein	15.3 ± 2.2	15.1 ± 2.2	15.4 ± 2.2	0.01
Fat	34.8 ± 5.6	35.1 ± 5.6	34.6 ± 5.6	0.09
Total alcohol, g/day	6.4 (1.2-16.6)	2.7 (0.006–9.2)	8.9 (2.7–17.8)	<0.001
Alcohol use per day				<0.001
no drink, *n* (%)	256 (16.0)	165 (28.7)	91 (12.0)	
≤1 drink/day, *n* (%)	572 (35.6)	262 (45.6)	310 (41.0)	
1-2 drinks/day, *n* (%)	323 (20.1)	105 (18.3)	218 (28.8)	
>2 drinks/day, *n* (%)	181 (11.3)	43 (7.5)	138 (18.2)	
Heavy drinker, *n* (%)	296 (18.4)	73 (12.7)	223 (29.5)	
Vitamin Supplementation use, *n* (%)	236 (14.7)	91 (11.3)	145 (18.1)	0.3
**Serum biomarkers**				
Glucose, mmol/L	5.2 (4.8-5.7)	5.3 (4.9–5.9)	5.1 (4.8–5.6)	<0.001
HbA1C %	5.9 ± 0.6	5.9 ± 0.6	5.8 ± 0.5	<0.001
Homocysteine, umol/L	13.0 (11.0–15.0)	14.0 (12.0–16.0)	13 (11–15)	<0.001
Folic acid, nmol/L	16.6 (11.1–24.9)	14.4 (9.7–22.7)	18.4 (12.7–27.4)	<0.001
Deficiency, *n* (%)	313 (19.5)	214 (27.8)	99 (12.9)	<0.001
Insufficiency, *n* (%)	262 (16.3)	144 (18.7)	118 (15.4)	
Sufficiency, *n* (%)	963 (60.0)	412 (53.5)	551 (71.7)	
Vitamin K, pmol/L	209.0 (136.9–291.2)	200.5 (135.7–287.5)	213.9 (138.3–296.6)	0.3
Deficiency, *n* (%)	105 (6.5)	46 (5.8)	59 (7.4)	0.2
Sufficiency, *n* (%)	1487 (92.6)	749 (94.2)	738 (92.6)	
Vitamin B12, pmol/L	290 (224.0–362.0)	275 (218–347.3)	303 (233.5–379.5)	<0.001
deficiency, *n* (%)	67 (4.2)	37 (4.8)	30 (3.9)	0.07
insufficiency, *n* (%)	295 (18.4)	162 (21.1)	133 (17.2)	
Sufficiency, *n* (%)	1177 (73.3)	567 (74.0)	610 (78.9)	
Vitamin B6, nmol/L	53.9 (36.9–81.3)	48.8 (34.0–72.9)	60 (40.7–91.2)	<0.001
deficiency, *n* (%)	65 (4.0)	48 (6.0)	17 (2.1)	<0.001
insufficiency, *n* (%)	178 (11.1)	108 (13.6)	70 (8.8)	
Sufficiency, *n* (%)	1350 (84.1)	638 (80.4)	712 (89.1)	
Vitamin E, umol/L	33.4 (29.3–38.5)	33.2 (28.4–38.1)	33.6 (29.7–38.9)	0.06
Deficiency, *n* (%)	0 (0)	0 (0)	0 (0)	
Insufficiency, *n* (%)	465 (29)	256 (32.1)	209 (26.2)	0.01
Sufficiency, *n* (%)	1131 (70.5)	542 (67.9)	589 (73.8)	
Vitamin A, umol/L	2.1 ± 0.5	2.1 ± 0.4	2.1 ± 0.5	0.09
Deficiency, *n* (%)	0 (0)	0 (0)	0 (0)	
insufficiency, *n* (%)	7 (0.4)	3 (0.4)	4 (0.5)	0.7
Sufficiency, *n* (%)	1562 (97.3)	774 (99.6)	788 (99.5)	
Vitamin D, nmol/L	63.2 ± 22.1	62.9 ± 21.3	63.5 ± 22.8	0.6
deficiency, *n* (%)	76 (4.7)	36 (4.8)	40 (5.3)	0.9
insufficiency, *n* (%)	369 (23.0)	184 (24.6)	185 (24.4)	
Sufficiency, *n* (%)	1060 (66.0)	528 (70.6)	532 (70.3)	
**Multivitamin status**				
Single vitamin deficiency, *n* (%)	460 (28.7)	265 (33.0)	195 (24.3)	<0.001
Single vitamin insufficienciy, *n* (%)	894 (55.7)	460 (57.3)	434 (54.1)	0.006
2 deficiencies, *n* (%)	89 (5.5)	58 (7.2)	31 (3.9)	0.001
2 insufficiencies, *n* (%)	397 (24.7)	216 (26.9)	181 (22.6)	0.008
>=3 deficiencies, *n* (%)	18 (1.1)	16 (2.0)	2 (0.2)	0.001
>=3 insufficiencies, *n* (%)	111 (6.9)	65 (8.1)	46 (5.7)	0.03

Abbreviations: SES: socioeconomic status, BMI: body mass index. The cutoff values applied for folic acid status, were serum folic acid < 10.2 nmol/L, 10.2–13.6 nmol/L, and > 13.6 nmol/L for deficiency, insufficiency, and sufficient status, respectively. Vitamin K insufficiency was defined as desphospho-uncarboxylated matrix Gla protein (dp-ucMGP) level higher than 500 pmol/L. The cutoff values that employed for vitamin B12 status were serum vitamin B12 < 150 pmol/L, 150–220 pmol/L, and > 220pmol/L for deficiency, insufficiency, and sufficient status, respectively. The cutoff values used for serum vitamin B6 status were serum vitamin B6 < 20 nmol/L, 20–30 nmol/L, and > 30 nmol/L for deficiency, insufficiency, and sufficient status, respectively. Serum alpha-tocopherol levels below 30 μmol/L were considered indicating vitamin E insufficiency. Vitamin A insufficiency was defined as serum retinol concentration below 0.7 μmol/L. The cutoff values applied for vitamin D status, were serum 25-hydroxyvitamin D (25(OH)D) < 30 nmol/L, 30–50 nmol/L, and > 50 nmol/L for deficiency, insufficiency, and sufficient status, respectively.

**Table 3:** Associations between socioeconomic status (SES) and vitamin deficiencies and insufficiencies * (High SES = reference) ^a^.

	Folic Acid		Vitamin B12		Vitamin B6		Vitamin D		Vitamin E		Vitamin K		Single Vitamin		Multivitamin	
	OR [95% CI]	*p*	OR [95% CI]	*p*	OR [95% CI]	*p*	OR [95% CI]	*p*	OR [95% CI]	*p*	OR [95% CI]	*p*	OR [95% CI]	*p*	OR [95% CI]	*p*
Model 1 ^b^																
Deficiency	**2.89 [2.21–3.79]**	<0.001	1.33 [0.81–2.18]	0.3	**3.15 [1.79–5.54]**	<0.001	0.91 [0.57–1.45]	0.7	/		0.77 [0.51–1.14]	0.2	**1.71 [1.36–2.14]**	<0.001	**2.06 [1.31–3.23]**	0.002
Insufficiency	**1.63 [1.24–2.15]**	<0.001	**1.31 [1.01–1.69]**	0.04	**1.72 [1.25–2.37]**	0.001	1.00 [0.79–1.27]	0.9	**1.33 [1.07–1.65]**	0.01	/		**/**		/	
Sufficiency	1 (ref)		1 (ref)		1 (ref)		1 (ref)		1 (ref)		1 (ref)					
Model 2																
Deficiency	**2.88 [2.18–3.80]**	<0.001	1.30 [0.78–2.16]	0.3	**2.99 [1.68–5.32]**	<0.001	0.85 [0.53–1.38]	0.5	/		0.75 [0.50–1.13]	0.2	**1.65 [1.30–2.08]**	<0.001	**1.96 [1.24–3.12]**	0.004
Insufficiency	**1.69 [1.28–2.25]**	<0.001	1.24 [0.95–1.62]	0.1	**1.60 [1.15–2.22]**	0.005	0.99 [0.77–1.26]	0.9	**1.33[1.06–1.66]**	0.01	/		**/**		/	
Sufficiency	1 (ref)		1 (ref)		1 (ref)		1 (ref)		1 (ref)		1 (ref)					
Model 3																
Deficiency	**2.72 [2.03–3.65]**	<0.001	1.61 [0.94–2.75]	0.09	**2.92 [1.59–5.36]**	0.001	0.72 [0.43–1.20]	0.2	/		0.70 [0.45–1.08]	0.1	**1.57 [1.22–2.01]**	<0.001	**1.79 [1.10–2.93]**	0.02
Insufficiency	**1.62 [1.20–2.19]**	0.002	1.31 [0.99–1.73]	0.06	1.37 [0.97–1.94]	0.07	0.86 [0.66–1.11]	0.2	**1.30 [1.03–1.65]**	0.03	/		**/**		/	
Sufficiency	1 (ref)		1 (ref)		1 (ref)		1 (ref)		1 (ref)		1 (ref)					
Model 4																
Deficiency	**2.46 [1.78–3.40]**	<0.001	1.29 [0.70–2.36]	0.4	1.63 [0.78–3.38]	0.2	**0.49 [0.26–0.93]**	0.03	/		0.74 [0.46–1.19]	0.2	**1.39 [1.06–1.84]**	0.02	1.28 [0.74–2.21]	0.4
Insufficiency	1.41 [1.01–1.97]	0.05	1.16 [0.85–1.59]	0.4	1.23 [0.84–1.81]	0.3	0.82 [0.61–1.11]	0.2	1.31 [1.00–1.71]	0.05	/		**/**		/	
Sufficiency	1 (ref)		1 (ref)		1 (ref)		1 (ref)		1 (ref)		1 (ref)					
Model 5																
Deficiency	**2.58 [1.87–3.57]**	<0.001	1.32 [0.72–2.40]	0.4	1.63 [0.79–3.38]	0.2	**0.48 [0.26–0.90]**	0.02	/		0.73 [0.45–1.16]	0.2	**1.40 [1.06–1.84]**	**0.02**	1.35 [0.78–2.34]	0.3
Insufficiency	**1.49 [1.07–2.08]**	0.02	1.14 [0.83–1.57]	0.4	1.34 [0.92–1.97]	0.1	0.85 [0.64–1.15]	0.3	1.28 [1.00–1.66]	0.07	/		**/**		/	
Sufficiency	1 (ref)		1 (ref)		1 (ref)		1 (ref)		1 (ref)		1 (ref)					
Model 6																
Deficiency	**2.55 [1.85–3.52]**	<0.001	1.65 [0.58–4.74]	0.3	1.76 [0.85–3.62]	0.13	**0.46 [0.25–0.86]**	0.02	/		0.76 [0.48–1.22]	0.3	**1.40 [1.06–1.84]**	0.02	1.25 [0.72–2.17]	0.4
Insufficiency	**1.50 [1.08–2.09]**	0.02	1.20 [0.88–1.64]	0.3	1.32 [0.91–1.95]	0.1	0.84 [0.63–1.12]	0.2	1.28 [0.98–1.67]	0.07	/		/		/	
Sufficiency	1 (ref)		1 (ref)		1 (ref)		1 (ref)		1 (ref)		1 (ref)					

Abbreviations: OR: odds ratio, CI: confidence interval, SES: socioeconomic status, ref: reference, LLDS: lifelines diet score. ^a^ Odds ratio with 95% CI and *p*-value were shown, *p* < 0.05 was considered significant and were shown in bold; vitamin A was not included because there were too few (*N* = 7) individuals who had an insufficient vitamin A status. ^b^ Model 1: unadjusted crude model with SES as independent variable; Model 2: model 1, additionally adjusted for age and gender; Model 3: model 2, additionally adjusted for BMI and smoking behavior; Model 4: model 3, additionally adjusted for the LLDS; Model 5: model 3, additionally adjusted for vegetable intake per day per 1000 kcal; Model 6: model 3, additionally adjusted for fruit intake per day per 1000 kcal. /: not applicable because of a lack of participants or cutoff points. * The cutoff values applied for folic acid status were serum folic acid < 10.2 nmol/L, 10.2–13.6 nmol/L, and > 13.6 nmol/L for deficiency, insufficiency, and sufficient status, respectively. Vitamin K insufficiency was defined as a desphospho-uncarboxylated matrix Gla protein (dp-ucMGP) level higher than 500 pmol/L. The cutoff values employed for vitamin B12 status, were serum vitamin B12 < 150 pmol/L, 150–220 pmol/L, and > 220 pmol/L for deficiency, insufficiency, and sufficient status, respectively. The cutoff values used for serum vitamin B6 status, were serum vitamin B6 < 20 nmol/L, 20–30 nmol/L, and > 30 nmol/L for deficiency, insufficiency, and sufficient status, respectively. Serum alpha-tocopherol levels below 30 μmol/L were considered indicating vitamin E insufficiency. Vitamin A insufficiency was defined as a serum retinol concentration below 0.7 μmol/L. The cutoff values applied for vitamin D status were serum 25(OH)D < 30 nmol/L, 30–50 nmol/L, and > 50nmol/L for deficiency, insufficiency, and sufficient status, respectively.

**Table 4:** Mediating the role of the LLDS on the associations between SES and vitamin deficiencies and insufficiencies **^a.^**

Predictor: Low SES ^b^ Mediator: LLDS	a Path		b Path		Mediation Effect		Total Effect		Prop. Mediated ^c^
	a (SE)	*p*	b (SE)	*p*	m (SE)	*p*	t (SE)	*p*	
**Single-vitamin deficiency**	−2.26 (0.37)	<0.001	−0.007 (0.007)	0.3	0.017 (0.016)	0.3	0.22 (0.09)	0.01	-
Multi-vitamin deficiency	−2.26 (0.40)	<0.001	−0.019 (0.011)	0.09	0.043 (0.027)	0.1	0.16 (0.13)	0.3	-
Folic acid deficiency	−2.35 (0.35)	<0.001	−0.022 (0.007)	0.002	0.051 (0.018)	0.004	0.51 (0.09)	<0.001	10.1%
Folic acid insufficiency	−2.35 (0.36)	<0.001	−0.018 (0.007)	0.01	0.043 (0.020)	0.03	0.11 (0.091)	0.2	39.1%
Vit B12 deficiency	−2.35 (0.36)	<0.001	−0.006 (0.013)	0.6	0.015 (0.030)	0.6	0.12 (0.14)	0.4	-
Vit B12 insufficiency	−2.34 (0.35)	<0.001	−0.018 (0.008)	0.02	0.041 (0.019)	0.03	0.11 (0.09)	0.2	37.2%
Vit B6 deficiency	−2.39 (0.35)	<0.001	−0.019 (0.013)	0.2	0.045 (0.033)	0.2	0.22 (0.18)	0.2	-
Vit B6 insufficiency	−2.39 (0.35)	<0.001	−0.022 (0.008)	0.006	0.052 (0.021)	0.01	0.14 (0.10)	0.1	37.1%
Vit D deficiency	−2.31 (0.36)	<0.001	0.009 (0.012)	0.5	−0.021 (0.030)	0.5	−0.32 (0.14)	0.03	-
Vit D insufficiency	−2.31 (0.36)	<0.001	−0.011 (0.007)	0.1	0.025 (0.017)	0.1	−0.07 (0.09)	0.5	-
Vit E insufficiency	−2.39 (0.36)	<0.001	0.004 (0.007)	0.5	−0.010 (0.016)	0.5	0.15 (0.08)	0.07	-
Vit K deficiency	−2.36 (0.33)	<0.001	−0.000 (0.009)	0.9	0.000 (0.022)	0.9	−0.14 (0.12)	0.2	-

Abbreviations: LLDS: Lifelines diet score, SE: standard error, SES: socioeconomic status, Prop.: mediated proportion mediated. ^a^ all analyses were adjusted for age, gender, BMI and smoking behavior, a, b, m, and t represented the estimates of different paths in the mediation model, standard error of the estimates and *p*-value were also shown. *p* < 0.05 was considered significant. vitamin A was not included because there were too few (*N* = 7) individuals who had an insufficient vitamin A status. The cutoff values applied for folic acid status were serum folic acid < 10.2 nmol/L, 10.2–13.6 nmol/L, and > 13.6 nmol/L for deficiency, insufficiency, and sufficient status, respectively. Vitamin K insufficiency was defined as desphospho-uncarboxylated matrix Gla protein (dp-ucMGP) level higher than 500 pmol/L. The cutoff values employed for vitamin B12 status were serum vitamin B12 < 150 pmol/L, 150–220 pmol/L, and > 220pmol/L for deficiency, insufficiency, and sufficient status, respectively. The cutoff values used for serum vitamin B6 status were serum vitamin B6 < 20 nmol/L, 20-30 nmol/L, and > 30 nmol/L for deficiency, insufficiency, and sufficient status, respectively. Serum alpha-tocopherol levels below 30 μmol/L were considered indicating vitamin E insufficiency. Vitamin A insufficiency was defined as serum retinol concentration below 0.7 μmol/L. The cutoff values applied for vitamin D status were serum 25(OH)D < 30 nmol/L, 30–50 nmol/L, and > 50nmol/L for deficiency, insufficiency, and sufficient status, respectively. ^b^ High SES was treated as a reference group. ^c^ proportion mediated was not calculated if the mediation effect was not significant.

## Supplementary Materials

The following are available online at https://www.mdpi.com/2072-6643/12/9/2659/s1, Figure S1: Flow chart of the participants in the study, Table S1: Lifelines Diet Score food groups consumption between low and high SES groups, Table S2: Association between SES and serum vitamin biomarkers concentration, Table S3: Mediating role of the LLDS on the association between SES and serum vitamin biomarker concentrations.
