# Supplementary material for: Vitamin Status and Diet in Elderly with Low and High Socioeconomic Status: The Lifelines-MINUTHE Study
Source: Nutrients. 2020 Aug 31;12(9):2659. doi: 10.3390/nu12092659 (PMC7551314; doi:10.3390/nu12092659)
Supplement: Supplementary file 1 [file nutrients-12-02659-s001.pdf]

Supplementary Materials:

Supplementary Table 1. Lifelines Diet Score food groups consumption between low and high SES groups

**Food groups**	Total	Low SES	High SES	p-value
**Positive food groups (gram/1000kcal/day)**				
Vegetables	56.8 (38.0-78.1)	52.6 (33.9-71.9)	60.5 (41.1-84.2)	<0.001
Fruit	100.0 (48.8-149.6)	90.4 (44.1-141.2)	108.3 (55.8-154.3)	0.001
Whole grain products	54.3 (38.3-69.7)	56.1 (43.7-70.8)	52.2 (35.1-68.4)	<0.001
Legumes and nuts	10.8 (5.1-18.1)	9.1 (4.4-16.3)	11.9 (5.8-19.5)	<0.001
Fish	7.1 (3.5-11.7)	5.5 (2.1-8.7)	8.3 (4.9-15.8)	<0.001
Oil and soft margarines	3.9 (1.3-11.8)	7.2 (1.4-14.6)	3.1 (1.2-9.0)	<0.001
Unsweetened dairy	85.8 (37.0-147.5)	79.2 (33.8-129.4)	89.4 (39.9-57.6)	0.007
Coffee	231.6 (157.9-314.2)	246.2 (165.1-331.2)	221.7 (155.3-298.4)	0.002
Tea	127.7 (41.5-231.0)	103.2 (17.3-192.9)	144.3 (58.8-255.2)	<0.001
**Negative food groups (gram/1000kcal/day)**				
Red and processed meats	28.0 (18.3-38.4)	30.9 (21.9-40.8)	25.7 (14.3-36.6)	<0.001
Butter and hard margarines	13.4 (7.4-22.6)	16.7 (9.6-26.4)	11.7 (5.6-18.9)	<0.001
Sugar-sweetened beverages	22.0 (3.7-62.1)	20.8 (0.0-60.3)	23.2 (5.3-64.1)	0.2
**Neutral food group (gram/1000kcal/day)**				
Eggs	6.4 (3.3-10.7)	6.9 (3.4-10.8)	6.0 (3.3-10.7)	0.2
**Undefined food groups (gram/1000kcal/day)**				
Potatoes	44.2 (25.7-62.3)	56.2 (38.4-74.4)	34.1 (19.6-53.0)	<0.001
Refined grain products	34.9 (22.3-50.1)	34.1 (20.3-53.0)	35.6 (23.5-49.1)	0.6
White, unprocessed meat	6.1 (3.4-9.2)	6.5 (3.8-9.2)	5.9 (3.0-9.2)	0.02
Cheese	15.8 (9.3-23.9)	14.0 (7.8-22.3)	17.3 (10.6-25.0)	<0.001
Savory and Ready products	17.1 (7.9-29.8)	12.0 (5.2-22.9)	22.1 (10.2-35.6)	<0.001
Sugary products	29.5 (19.3-42.6)	30.9 (21.1-43.9)	28.3 (17.6-41.1)	0.002
Soups	18.7 (10.6-37.8)	18.9 (11.2-36.7)	18.3 (10.1-38.8)	0.9
Sweetened dairy products	47.8 (20.6-77.0)	54.7 (30.7-80.3)	41.7 (15.7-75.7)	<0.001
Artificially sweetened products	0.0 (0.0-16.2)	0.0 (0.0-24.7)	0.0 (0.0-11.6)	0.001

Abbreviations: *SES* socioeconomic status ; $p<0.05$ was considered significant. The intake of food groups was described as median (interquartile range [IQR]).

Supplementary Table 2. Association between SES and serum vitamin biomarkers concentration [a]

	Folic acid		Vitamin B12		Vitamin B6		Vitamin D		Alpha tocopherol		Retinol		Vitamin K	
	standardized β (se)	p	standardized β (se)	p	standardized β (se)	p	standardized β (se)	p	standardized β (se)	p	standardized β (se)	p	standardized β (se)	p
Model 1 [b]														
Low versus high SES	-0.17 (0.52)	<0.001	-0.06 (8.10)	0.01	-0.11 (4.18)	<0.001	-0.01 (1.14)	0.6	-0.03 (0.39)	0.3	-0.04 (0.02)	0.09	-0.03 (9.47)	0.2
Model 2														
Low versus high SES	-0.18 (0.53)	<0.001	-0.07 (8.27)	0.006	-0.11 (4.29)	<0.001	-0.01 (1.18)	0.7	-0.01 (0.40)	0.6	-0.04 (0.02)	0.1	-0.03 (9.76)	0.2
Model 3														
Low versus high SES	-0.17 (0.56)	<0.001	-0.07 (8.76)	0.007	-0.09 (4.49)	0.001	0.03 (1.23)	0.2	-0.01 (0.43)	0.8	-0.06 (0.03)	0.03	-0.03 (10.39)	0.1
Model 4														
Low versus high SES	-0.14 (0.63)	<0.001	-0.06(9.36)	0.05	-0.06 (5.19)	0.05	0.06 (1.38)	0.07	-0.01 (0.48)	0.7	-0.06 (0.028)	0.03	-0.03 (12.26)	0.1
Model 5														
Low versus high SES	-0.14 (0.63)	<0.001	-0.06 (9.31)	0.07	-0.06 (5.15)	0.04	0.05 (1.38)	0.1	-0.01 (0.47)	0.9	-0.05 (0.03)	0.1	-0.03 (12.19)	0.3
Model 6														
Low versus high SES	-0.14 (0.62)	<0.001	-0.06 (9.25)	0.04	-0.07 (5.13)	0.03	0.06 (1.37)	0.04	-0.01 (0.47)	0.8	-0.05 (0.03)	0.09	-0.03 (12.13)	0.3

Abbreviations: *se* standardized error, *SES* socioeconomic status; Vitamin D and vitamin K concentrations were measured as 25 hydroxyvitamin D (25 (OH)D) and desphospho-uncarboxylated matrix Gla protein (dp-ucMGP) levels, respectively.-

[a]Standardized coefficient with standardized error and p value were shown from multiple linear regression

[b]Model 1: unadjusted crude model with SES as independent variable; Model 2: model1, additionally adjusted for age and gender; Model 3: model 2, additionally adjusted for BMI and smoking behavior; Model 4: model3, additionally adjusted for the LLDS; Model 5: model 3, additionally adjusted for vegetable intake per day per 1000 kcal; Model 6: model 3, additionally adjusted for fruit intake per day per 1000 kcal.

Supplementary Table 3. Mediating role of the LLDS on the association between SES and serum vitamin biomarker concentrations [a]

Predictor: SES low [b] Mediator: LLDS	a path		b path		Mediation effect		Total effect		Prop. mediated [c]
	a (se)	p	b(se)	p	m (se)	p	t (se)	p	
Folic acid	-2.35 (0.36)	<0.001	0.20 (0.05)	<0.001	**-0.47 (0.14)**	0.001	-3.29 (0.60)	<0.001	**14.3%**
Vitamin B12	-2.35 (0.35)	<0.001	1.19 (0.71)	0.1	-2.79 (1.75)	0.1	-21.61 (9.12)	0.02	-
Vitamin B6	-2.39 (0.35)	<0.001	0.89 (0.38)	0.02	**-2.12 (0.94)**	0.03	-12.30 (4.91)	0.01	**17.2%**
Vitamin D	-2.31 (0.36)	<0.001	0.029 (0.11)	0.8	-0.068 (0.27)	0.8	2.41 (1.28)	0.06	-
Vitamin E	-2.39 (0.36)	<0.001	-0.041 (0.04)	0.3	0.10 (0.10)	0.4	-0.10 (0.50)	0.8	-
Vitamin A	-2.39 (0.36)	<0.001	-0.005 (0.002)	0.04	**0.013 (0.006)**	0.04	-0.047 (0.030)	0.1	**27.7%**
Vitamin K	-2.39 (0.36)	<0.001	-0.88 (1.12)	0.4	2.10 (2.68)	0.4	-20.39 (14.56)	0.2	-

Abbreviations: *LLDS* Lifelines diet score, *se* standard error, *SES* socioeconomic status, *Prop.mediated* proportion mediated; Vitamin D and vitamin K concentrations were measured as 25 hydroxyvitamin D (25 (OH)D) and desphospho-uncarboxylated matrix Gla protein (dp-ucMGP) levels, respectively. [a] all analyses were adjusted for age, gender, BMI and smoking behavior, a, b, m and t represented the estimates of different paths in the mediation model, standard error of the estimates and p value were also shown, p<0.05 was considered significant

[b] High SES was treated as a reference group

[c] proportion mediated was not calculated if the mediation effect was not significant

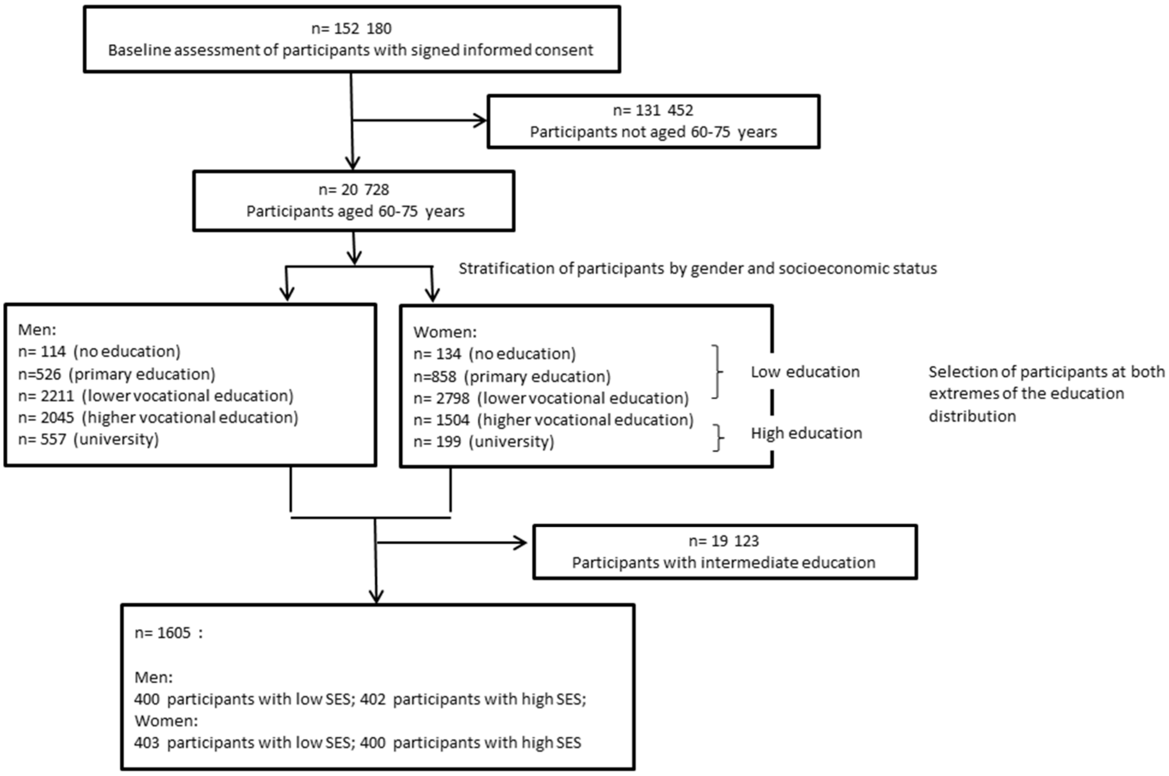

Supplementary Figure. 1. Flow chart of the participants in the study
